# Measuring patient-reported physical functioning and fatigue in myelodysplastic syndromes using a modular approach based on EORTC QLQ-C30

**DOI:** 10.1186/s41687-021-00334-w

**Published:** 2021-07-20

**Authors:** Antoine Regnault, Farrah Pompilus, Anna Ciesluk, Flora Mazerolle, Rafael Bejar, Robert J. Fram, Douglas V. Faller, Patrick Marquis, Jill A. Bell

**Affiliations:** 1 Modus Outcomes, 4 Place Amédée Bonnet, 69002 Lyon, France; 2 Modus Outcomes, Cambridge, MA USA; 3 Moores Cancer Center, UC San Diego – MDS Center of Excellence, San Diego, CA USA; 4 Millennium Pharmaceuticals, Inc., a wholly owned subsidiary of Takeda Pharmaceutical Company Limited, Cambridge, MA USA

**Keywords:** Patient-reported outcomes, Myelodysplastic syndromes, Acute myeloid leukemia, Chronic myelomonocytic leukemia, Fatigue, EORTC item library, Modular measurement approach, Physical functioning

## Abstract

**Purpose:**

Physical functioning and fatigue are key patient concerns in myelodysplastic syndromes (MDS), chronic myelomonocytic leukemia (CMML), and acute myeloid leukemia (AML). The objective of this research was to generate supportive quantitative evidence for modular physical functioning and fatigue measures based on the European Organisation for Research and Treatment of Cancer (EORTC) Quality of Life Questionnaire Core 30 items (QLQ-C30) and a customized selection of 10 supplemental items from the EORTC Item Library.

**Methods:**

The 40 items were completed online cross-sectionally by 51 patients (higher risk [HR] MDS: 53%; CMML: 26%; AML: 10%). Psychometric analyses based on Rasch measurement theory (RMT) were conducted on the QLQ-C30 physical functioning and fatigue domains as well as measures combining QLQ-C30 and supplemental items. A measure of anemia-related symptoms composed of QLQ-C30 and supplemental items covering fatigue, dyspnea, and dizziness was also investigated.

**Results:**

The QLQ-C30 physical functioning and fatigue domains showed good targeting to the sample and adequate reliability, with few conceptual gaps identified. Combining the QLQ-C30 and supplemental physical functioning and fatigue items improved the conceptual coverage and the reliability of the measures. The patient-reported anemia-related symptom measure showed good measurement performance, underpinned by a clinically meaningful characterization of severity of these symptoms over a spectrum, starting with fatigue, then dyspnea, and finally dizziness (most severe).

**Conclusion:**

The modular measurement approach of combining EORTC QLQ-C30 and Item Library offers a promising pragmatic solution to the measurement of physical functioning and fatigue, as well as anemia-related symptoms in clinical trials conducted in HR MDS, CMML, and AML.

**Supplementary Information:**

The online version contains supplementary material available at 10.1186/s41687-021-00334-w.

## Introduction

Myelodysplastic syndromes (MDS), chronic myelomonocytic leukemia (CMML), and acute myeloid leukemia (AML) are rare blood cancers that affect the myeloid cells. They are associated with anemia, neutropenia, and thrombocytopenia, which lead to a variety of symptoms and functional impacts for affected patients [1]. Measuring patient-reported outcomes (PROs) is key to understanding the patient experience in the field of hematology in both research and practice [2, 3]. Three core PRO concepts have been identified for oncology trials and should be measured to integrate the experience of patients and to demonstrate the benefit of new treatments as well as inform decision making: physical functioning, disease-related symptoms, and symptomatic adverse events [4]. The measurement of these core PRO concepts in oncology trials is increasingly performed using a modular approach. In a modular measurement approach, only the key concepts for a specific context of use are carefully selected and measured in the study, using items that are thoughtfully selected from existing static questionnaires and item banks or libraries [4, 5]. This approach allows a bespoke and efficient measurement of the concepts that are meaningful to patients.

Assessing symptomatic adverse events of a new treatment for MDS, CMML, and AML will be dependent on the treatment under consideration, as different treatments may have different safety profiles. Patient reporting of symptomatic adverse events would typically be done using the PRO-Common Terminology Criteria for Adverse Events (PRO-CTCAE) [6, 7], with a selection of PRO-CTCAE items defined on a case-by-case basis depending on the expected adverse event symptom profile of each treatment [8].

In contrast, a measurement strategy for physical functioning and disease-related symptoms in MDS, CMML, and AML can be achieved independently of the treatment under consideration. For this purpose, one must identify the specific, key concepts to measure and the most appropriate instrument to use to assess these symptoms and aspects of physical functioning in this context of use. Previous research identified the key concepts of importance for patients and organized them into a conceptual model for the measurement of PROs in higher risk (HR) MDS, CMML, and AML [9]. Fatigue was identified as a core symptom, as well as other anemia-related symptoms, such as dyspnea and dizziness.

A modular approach based on the European Organisation for Research and Treatment of Cancer (EORTC) Quality of Life Questionnaire-Core 30 items (QLQ-C30) and Item Library is most appropriate to measure physical functioning and core symptoms of HR MDS, CMMT and AML [9]. The EORTC QLQ-C30 alone already includes domain scores for physical functioning and core symptoms of HR MDS, CMML, and AML, such as fatigue, and has shown good measurement performance for this population [10]. A modular approach combining the QLQ-C30 and a customized selection of items from the EORTC Item Library allows bridging the conceptual gaps identified on the QLQ-C30 compared to the framework previously created for HR MDS, CMML, and AML. Ten items from the EORTC Item Library were identified as potential supplementary items to the QLQ-C30 to create a customized measure of key PROs to capture clinical benefit in HR MDS, CMML, and AML for inclusion in clinical trials. This modular approach also has the advantage of offering a pragmatic solution to the challenges associated with the measurement of PROs in rare diseases [11], such as MDS, AML, or CMML, as it builds on existing established material and does not require researchers to develop new, disease-specific PRO measures.

The objective of this research was to generate supportive quantitative evidence on the appropriateness of using the EORTC QLQ-C30 and 10 supplemental items from the EORTC Item Library to assess key PROs for people with HR MDS, CMML, and AML; specifically, physical functioning, fatigue, and other anemia-related symptoms.

## Methods

The items of the EORTC QLQ-C30 and 10 supplemental items chosen from the EORTC Item Library were compiled into an online survey. The survey was administered to people living with HR MDS, CMML, and AML at one time point.

### Patient sample

Patients were recruited using convenience sampling (non-probability sampling) through the MDS Foundation, social media, and market research agencies. Patients were included if they met all of the following criteria: were willing to consent; spoke, read, and understood English; were located in the US at the time of survey completion; were 18 years old or older; had a self-reported diagnosis of HR MDS, CMML, or AML; and had a patient-rated Eastern Cooperative Oncology Group (ECOG) status of 0, 1, or 2 [12]. Patients were excluded if they met any of the following criteria: were receiving daunorubicin or idarubicin; had participated in a clinical trial for treatments related to MDS, CMML, or AML within the previous 2 weeks; underwent major surgery within the previous 2 weeks; had a diagnosis or had been treated for another cancer in the previous 2 years; had a life-threatening illness unrelated to cancer; had a visual, cognitive, or linguistic impairment preventing them from understanding and answering the survey questions; or had a patient-rated ECOG status of 3 or 4.

Recruitment was stopped when a total of 50 participants completing the online survey was reached. Given the practical challenges of recruitment in a rare disease context, a sample of 50 patients was deemed sufficient to generate early evidence on the item sets under scrutiny using the anticipated methods, especially as the items included in the analyses were carefully selected to be appropriate to the context of use and were therefore expected to be well targeted to the sample.

### Ethics

All study documents and procedures were approved by an independent institutional review board (Copernicus Group IRB).

### EORTC QLQ-C30 and item library

The EORTC QLQ-C30 questionnaire is a standard PRO instrument in cancer patient populations [13]. It has five functional scales, eight symptom scales, a finance item, and a global health and Quality of Life (QoL) scale (Table 1). All items have four response choices, except the global health QoL scale, which has seven. QLQ-C30 scale scores range from 0 to 100. Higher scores represent better functioning/health status for the functioning scales and the global health status/QoL, but more severe symptoms for the symptom scales.

**Table 1:** Number of EORTC QLQ-C30 and supplemental items from the EORTC Item Library to cover key patient-relevant concepts in HR MDS, CMML, and AML

	EORTC QLQ-C30	EORTC Item Library
Global health status/QoL (QL)	2	
Physical functioning (PF)	5	2
Role functioning (RF)	2	2
Emotional functioning (EF)	4	
Social functioning (SF)	2	
Cognitive functioning (CF)	2	
Fatigue (FA)	3	3
Pain (PA)	2	
Dyspnea (DY)	1	2
Nausea/Vomiting (NV)	2	
Insomnia (SL)	1	
Appetite loss (AP)	1	
Constipation (CO)	1	
Diarrhea (DI)	1	
Financial difficulties (FI)	1	
Dizziness (DZ)		1

The EORTC Item Library is an online platform that compiles more than 900 individual items from over 60 EORTC questionnaires [14]. Users can select ad-hoc sets of items that were developed with the same general principles as the EORTC QLQ-C30; items have the same response options and recall period as the QLQ-C30 and, for some items, translations are already available in a number of languages.

In our research, a set of 10 items from the EORTC Item Library were identified based on the conceptual model resulting from previous qualitative research and testing [9] (Table 1). These items were selected as they were measuring concepts strongly endorsed by patients or considered a core symptom/impact by clinicians, were not primarily considered a side-effect of treatment, and could potentially capture a treatment benefit. These 10 items were shown to be clear and relevant in cognitive debriefing interviews with patients with HR MDS, CMML, or AML. Of note, in addition to the items added to measure physical functioning (Difficulty climbing stairs, Feeling slowed down), fatigue (Weakness in arms or legs, Becoming easily tired, Lacking energy), and other anemia-related symptoms (Shortness of breath on exertion, Having to stop for breath when walking and Dizziness), two items assessing role functioning were also selected to be tested as complementary to the original QLQ-C30 role functioning (RF) domain.

### Statistical analyses

Responses to the EORTC QLQ-C30 and 10 supplemental items from the EORTC Item Library were described. Psychometric analyses were performed in the Rasch measurement theory (RMT) framework. RMT uses a mathematical model, the Rasch model, to examine the legitimacy of creating scores from a set of items [15–17]. The following properties were explored in the RMT framework:

- Scale-to-sample targeting was assessed by visual inspection of the relative distribution of item locations and person measurements on their shared continuum.
- Adequacy of response scales was assessed by examining whether all ‘item thresholds’ (i.e., the point in the continuum where the most probable response between two adjacent response category changes) were properly ordered.
- Item fit was assessed by joint examination of statistical parameters (log residuals and chi-square values comparing between observed and expected responses to an item) and item characteristic curves (ICC) that graphically display the expected responses across the continuum of person scores and the observed values for each class interval of person scores. Fit residuals outside the recommended range of − 2.5 / 2.5 and statistically significant chi-square values are indicative of possible fit issues [17].
- Reliability was assessed using Person Separation Index (PSI). Reliability coefficients are interpreted as follows: < 0.70: Poor; 0.70–0.79: Modest; 0.80–0.89: Adequate; 0.90–0.94: Good; > 0.95: Excellent [18].
- Local item dependency was assessed by examining the correlations between the standardized item residuals. Any positive correlation greater than 0.3 was considered as possibly indicative of item dependency [17].

Two sets of items for physical functioning and fatigue were tested separately using the Rasch model: first, items from the EORTC QLQ-C30 PF and FA domains and then the combination of the same QLQ-C30 items with the supplementary items from the EORTC Item Library. All items reflecting patient-reported anemia-related symptoms (i.e. items pertaining to fatigue, dyspnea, and dizziness) were also tested together in a separate Rasch model. The Rasch model was also applied to the items pertaining to role functioning but the results of this analyses are not presented here.

The RMT analyses were performed using RUMM2030 (RUMM Laboratory; Perth, Australia). All other statistical analyses were performed using SAS v9.4 (SAS Institute; Cary, NC, USA).

## Results

### Patient demographics

A total of 51 patients participated in the online survey, with most patients diagnosed with HR MDS (HR MDS: 53%; CMML: 26%; AML: 10%). All patients confirmed a diagnosis of HR MDS, CMML, and AML to be included in the study. However, 6 patients did not report which was their exact diagnosis. The mean age was 68 years (SD: 12) and 49% of patients were female (Table 2).

**Table 2:** Sample description

	Total (***N*** = 51)
**Age** (in years)	
Mean (SD)	68 (12)
Median (Min – Max)	71 (26–84)
**Gender** – Female N (%)	25 (49)
**Employment status** – N (%)	
Working full-time	7 (14)
Working part-time	4 (8)
Retired	22 (43)
Other*	12 (24)
Missing	6 (12)
**Living situation**	
Lives alone	12 (24)
Lives in a nursing home or assisted living facility	1 (2)
Lives with a partner, spouse, family or friends	32 (63)
Missing	6 (12)
**Diagnosis** – N (%)	
MDS	27 (53)
CMML	13 (26)
AML	5 (10)
Missing**	6 (12)
**Number of years since diagnosis**)	
Mean (SD)	5.0 (7.4)
Median (Min – Max)	3 (0–46)
**Prognostic risk category** (IPSS-R)*** – N(%) in MDS only (Total *N* = 27)	
Very high (> 6 points)	1 (4)
High (> 4.5–6 points)	10 (37)
Intermediate (> 3–4.5 points)	3 (11)
Don’t know	13 (48)
**ECOG status** – N (%)	
0 – Fully active	7 (14)
1 – Restricted in physically strenuous activity but ambulatory	12 (24)
2 – Ambulatory and capable of all selfcare	4 (9)
Missing	28 (55)
**Stem cell transplant** – N (%)	1 (2)
Patients using supportive care therapies****	18 (35)

*Other employment status included student, not employed, retired-not employed, and disabled-not employed

**Missing diagnosis: patient with MDS, CMML, or AML who did not complete the question on the demographic and health information form

***Prognostic risk category only assessed for patients with HR MDS and CMML (n = 40)

****Examples of supportive care therapies include transfusions, granulocyte colony stimulating factor, granulocyte macrophage-colony stimulating factor, thrombopoietin, erythropoiesis stimulating agents, etc.

### Description of responses to EORTC QLQ-C30 and item library items

**Fig. 1:**
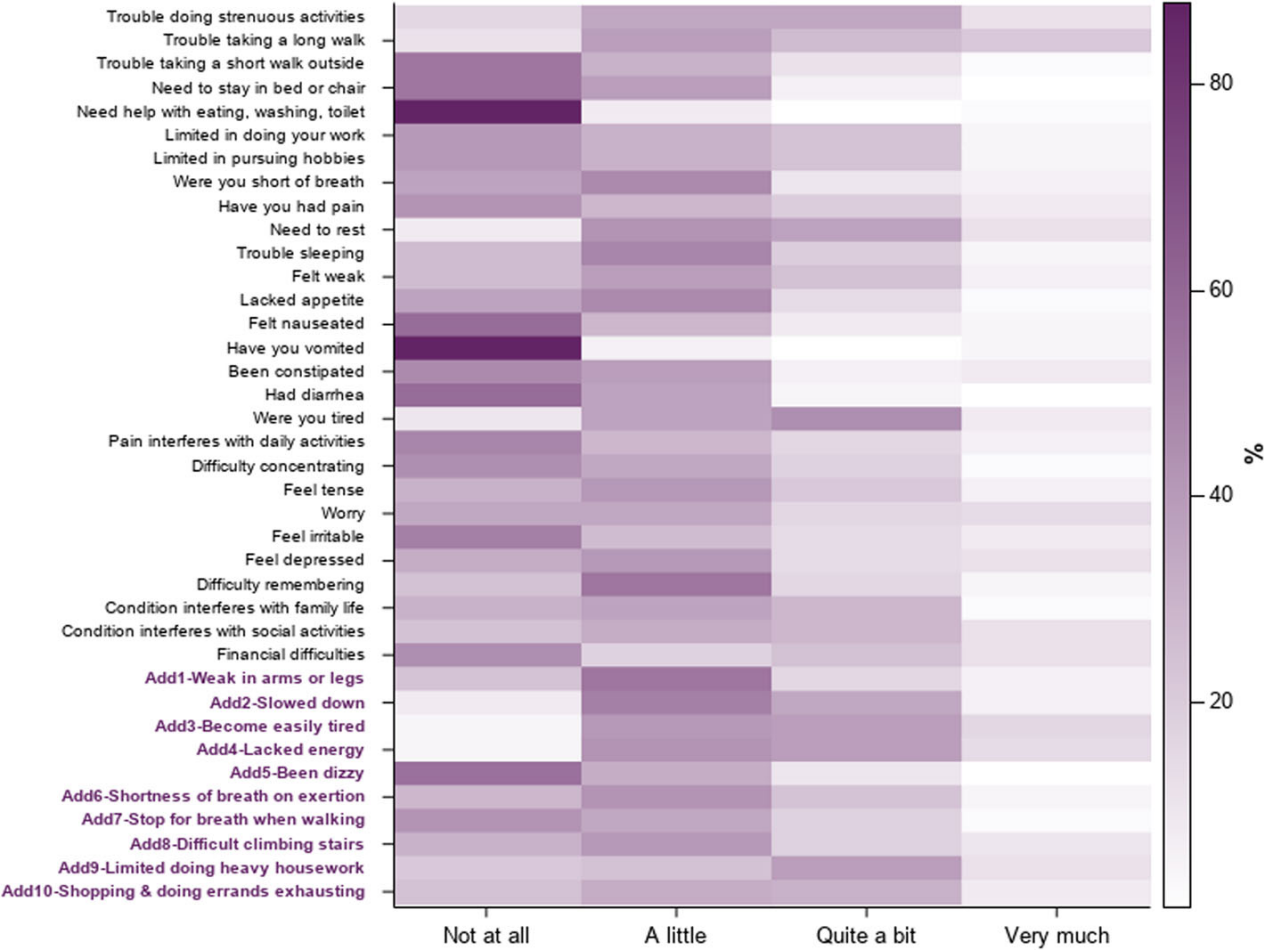
Distribution of the responses to the original EORTC QLQ-C30 items and supplemental items from the EORTC Item Library (*N* = 51). Darker colors indicate higher percentages of patients who endorsed the response. Items with bold purple labels are the 10 supplemental items from the EORTC Item Library

A possible floor effect for several EORTC QLQ-C30 items was identified (i.e., a substantial percentage of responders reported the lowest value [‘Not at all’]), whereas the responses to the supplemental items were well distributed across the response categories, with the exception of “dizziness” for which a greater number of patients responded ‘Not at all’ (Fig. 1). This confirmed that the customized supplemental item selection was well targeted to the patient sample overall.

### RMT analysis of physical functioning items

**Fig. 2:**
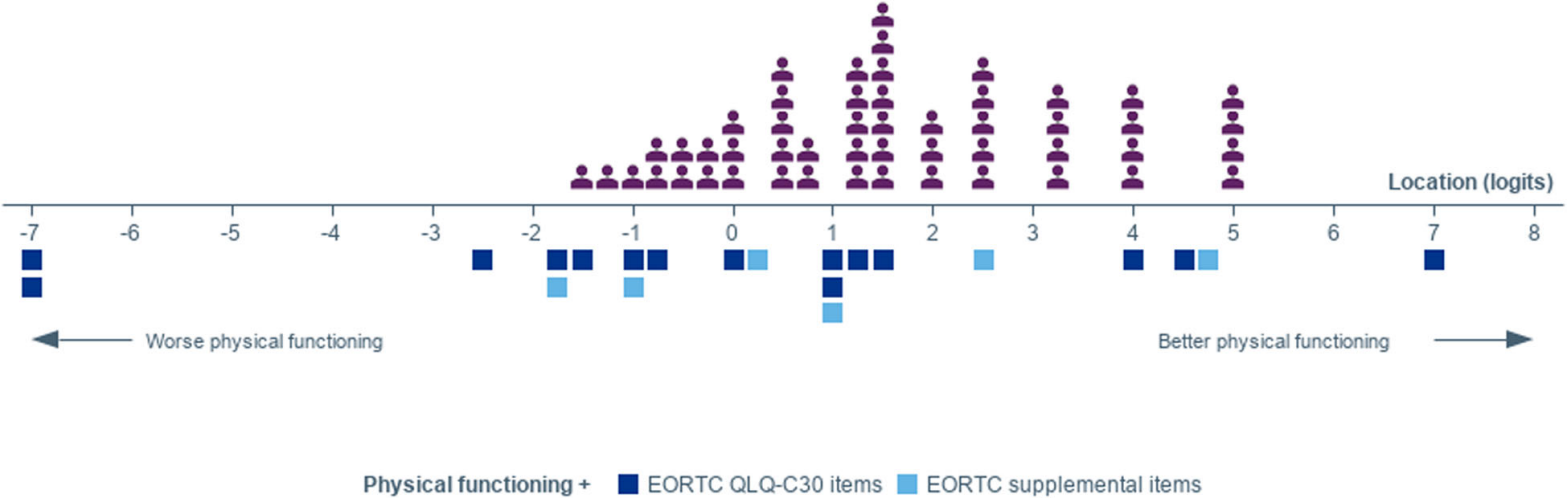
Scale to sample targeting of the original EORTC QLQ-C30 and supplemental Physical Functioning (PF) items (*N* = 51). The upper panel shows the distribution of the 51 individuals of the survey sample over the physical functioning continuum; the lower panel (blue squares) show the distribution of the ‘response thresholds’ (i.e., the point of the continuum where the most probable response between two adjacent response categories for an item changes) of the EORTC QLQ-C30 and supplemental physical functioning items on the physical functioning continuum

The EORTC QLQ-C30 PF items demonstrated acceptable measurement performance: good targeting of the items to the patient sample (Fig. 2), adequate reliability, fit of all items to the Rasch model, and intended function of response scales for all items but one (hygiene-related) (Table 3 and Supplementary material 1 and 2). No pairs of items showed high correlations in standardized residuals (see supplementary material 1). Some gaps in the coverage of the physical functioning continuum were uncovered; for example, few items provided an opportunity to differentiate between scores of patients with higher levels of physical functioning (Table 3).

**Table 3:** Summary results of the Rasch measurement theory analysis of 1) the EORTC QLQ-C30 PF items and 2) EORTC QLQ-C30 and supplemental PF items

	EORTC QLQ-C30 Physical functioning			EORTC QLQ-C30 Physical functioning and supplemental items		
	Disordered threshold	Fit residuals	PSI	Disordered threshold	Fit residuals	PSI
Trouble doing strenuous activities	No	− 0.771	0.82	No	− 0.939	0.83
Trouble taking a long walk	No	−1.767		No	−1.506	
Trouble doing short walk out	No	−0.692		No	−0.766	
Need to stay in bed or chair	No	2.334		No	1.841	
Need help with eat wash toilet	Yes	−0.93		Yes	− 0.829	
Slowed down				No	0.795	
Difficult climbing stairs				No	−0.291	

The addition of the two physical functioning items from the EORTC Item Library to the QLQ-C30 PF items bridged some gaps in the coverage of the functioning continuum, especially thresholds between 0 and 1 logit and between 2 and 4 logits (where no threshold was originally present) and at about 5 logits (Fig. 2). The new items therefore added information for a part of the physical functioning continuum where most patients where distributed. It also marginally improved reliability, without adding any issue in terms of fit, item dependency, or adequacy of the response options (Table 3 and Supplementary material 1).

### RMT analysis of fatigue and other anemia-related symptoms

**Fig. 3:**
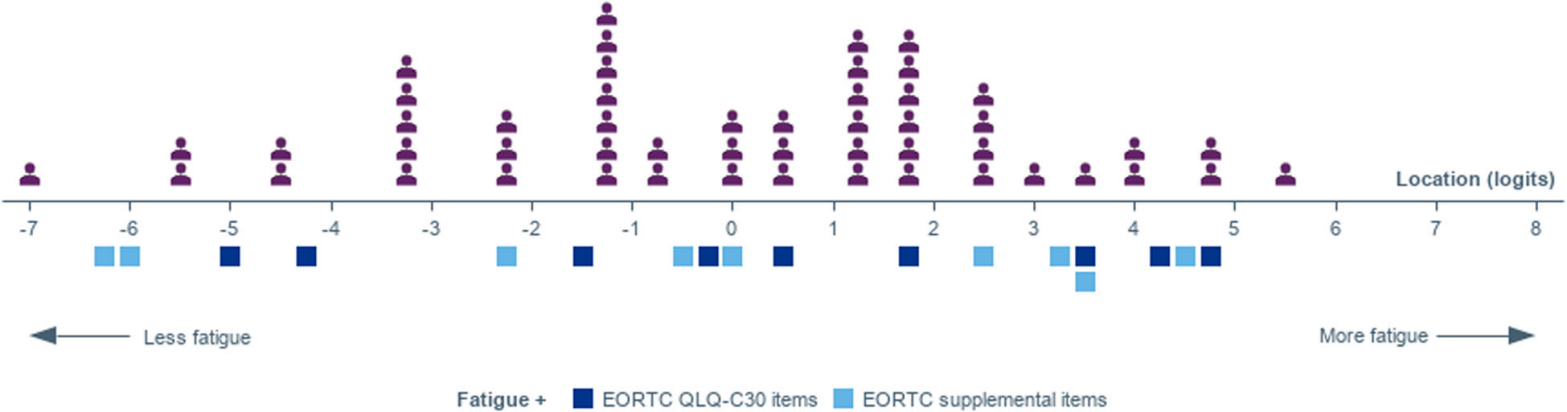
Scale to sample targeting of the original EORTC QLQ-C30 and supplemental Fatigue items (*N* = 51). The upper panel shows the distribution of the 51 individuals of the survey sample over the fatigue continuum; the lower panel (blue squares) show the distribution of the ‘thresholds(i.e., the point of the continuum where the most probable response between two adjacent response categories for an item changes)’ of the EORTC QLQ-C30 and supplemental fatigue items on the fatigue continuum

The EORTC QLQ-C30 FA items demonstrated acceptable measurement performance: good targeting of the items to the patient sample (Fig. 3), adequate reliability, fit of all items to the Rasch model, and intended functioning of all response scales (Table 4 and Supplementary material 1 and 2). No pairs of items showed high correlations in standardized residuals (see supplementary material 1). However, some gaps in the coverage of the fatigue continuum were uncovered (Fig. 3).

**Table 4:** Summary results of the Rasch measurement theory analysis of 1) the EORTC QLQ-C30 FA items, 2) the EORTC QLQ-C30 and supplemental FA items, and 3) the EORTC QLQ-C30 and supplemental items assessing fatigue and other anemia-related symptoms

	EORTC QLQ-C30 Fatigue			EORTC QLQ-C30 Fatigue and supplemental items			Fatigue and other anemia-related symptoms		
	Disordered threshold	Fit residuals	PSI	Disordered threshold	Fit residuals	PSI	Disordered threshold	Fit residuals	PSI
Need to rest	No	−0.375	0.82	No	0.515	0.90	No	−0.205	0.89
Felt weak	No	−0.171		No	0.462		No	−0.021	
Were you tired	No	0.242		No	0.159		No	−0.760	
Weak in arms or legs				No	0.233		No	0.244	
Become easily tired				No	−2.091		No	−1.171	
Lacked energy				No	−0.917		No	−0.506	
Were you short of breath							No	0.777	
Shortness breath on exertion							No	0.660	
Stop for breath when walking							No	1.357	
Been dizzy							No	0.777	

The addition of the three fatigue items from the EORTC Item Library to the QLQ-C30 FA items bridged some gaps in the coverage of the fatigue continuum (Table 4). It also improved reliability to a very good level, without adding any issue in terms of fit, or adequacy of the response options (Table 4). The items “Becoming easily tired” and “Lacking energy” showed some possible dependency (Standardized residual correlation: 0.45 – Supplementary material 1).

**Fig. 4:**
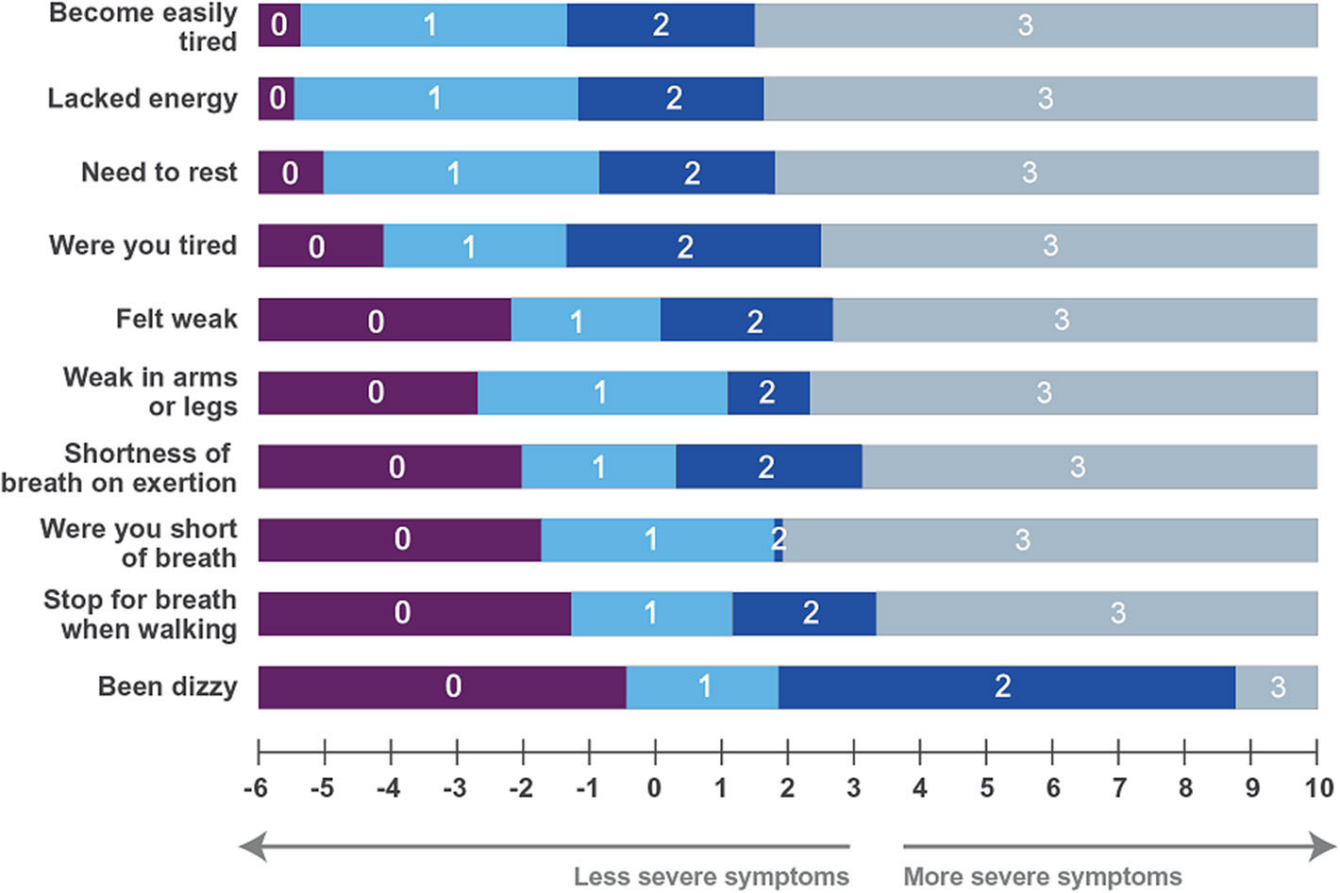
The EORTC QLQ-C30 and Item Library items delineates the progression of anemia-related symptom severity from fatigue to dyspnea and dizziness in HR MDS, CMML, and AML patients. Item threshold map from RMT analysis on online survey data (*N* = 51) For each item, the most probable response is presented depending on the position on the symptom severity continuum (0: Not at all; 1: A little; 2: Quite a bit; 3: Very much)

Combining the items assessing fatigue, dyspnea, and dizziness in a single scale intended to measure the severity of symptoms related to anemia among patients with HR MDS, CMML, and AML led to a measure with adequate measurement performances: good targeting (*data not shown*), good reliability, fit of all items to the Rasch model, and no issues in terms of response scales. Some possible dependency was found between three pairs of items (“Becoming easily tired” and “Lacking energy” (0.69); “Shortness of breath” and “Shortness of breath on exertion” (0.65); “Become easily tired” and “Weakness in arms or legs” (0.35)– Supplementary material 1). Most importantly, the RMT analysis uncovered a meaningful hierarchy of the anemia-related symptom items, in which fatigue comes first, followed by dyspnea, and dizziness (Fig. 4).

### Proposed new scores specific to HR MDS, CMML and AML using EORTC QLQ-C30 and supplemental items

The RMT analyses warrant the creation of scores combining the items from the QLQ-C30 and Item Library for PF, FA, and a combination of all items assessing fatigue and other symptoms related to anemia. For consistency, we recommend that the calculation of these scores follow the same principles as the calculation of the QLQ-C30 scores, keeping a range from 0 to 100.

## Discussion

In this research, we created customized PRO measures of physical functioning and fatigue for HR MDS, CMML, and AML using items from the EORTC QLQ-C30 and Item Library. We also explored a wider measure targeting the symptomatic manifestations of anemia, a core clinical feature of HR MDS, CMML, and AML, based on 10 PRO items covering fatigue plus dyspnea and dizziness. Quantitative evidence from the application of modern psychometrics in a sample of 51 patients supported the appropriateness of the customized measures in this context.

Our modular measurement approach to the key PROs in HR MDS, CMML, and AML was conceptually driven. We first conducted sound qualitative research including a literature review, interviews with clinicians and patients, and the development of a conceptual model summarizing the key outcomes for patients with MDS, CMML, and AML [9]. We then used the EORTC Item Library to complement the QLQ-C30 based on the conceptual model. The analyses described here generated supportive evidence of the relevance of the modular measurement approach previously described and available by using the EORTC Item Library as a resource to supplement legacy EORTC instrument; our research show how this method ensures that PROs are well suited for specific contexts of use like rare cancers. We showed that the EORTC QLQ-C30 scores had acceptable performance in patients with HR MDS, CMML, and AML and that the supplemental items selected from the EORTC Item Library improved them. The conceptual clarity made possible by the EORTC QLQ-C30 and supplemental items is illustrated by the clinically meaningful ordering of anemia-related symptoms uncovered by the RMT analysis of these data: it showed that the perception of symptoms of anemia by patients with HR MDS, CMML and AML appears to start by fatigue (first fatigability, then weakness) then dyspnea (first on exercise, then at rest), and finally dizziness. The positive results of our RMT analyses for the various item sets that we investigated and the informative findings from our analyses is certainly stemming from the early thoughtful conceptualization and qualitative work conducted with patients to carefully craft the item sets that are used to measure the concepts of interest in the specific context of the research. Thus, we would recommend that any modular measurement research endeavor put emphasis on these initial stages.

Our research focused on the measurement of core outcomes for patients with HR MDS, CMML, or AML, namely physical functioning, fatigue, and anemia-related symptoms. Yet, other concepts are relevant to capture the full patient experience in this context. First, role functioning is recognized as an important PRO concept and was identified in our conceptual model. However, while we added two items from the EORTC Item Library based on our predefined conceptualization of patient experience with MDS, CMML, and AML to the two QLQ-C30 RF items, we were not able to demonstrate that the resulting item set had strong measurement properties. Including more role functioning items, for example from the 10-item bank used for the EORTC role functioning computer adaptive test [19], may be an option to create a measure of role functioning with better measurement properties based on the EORTC measurement system. Besides, PRO instruments such as the QOL-E [20] and the Quality of Life in Myelodysplasia Scale (QUALMS) [21] have been developed to measure more distal concepts, such as health-related quality of life, in MDS. The possibility that these instruments may be relevant to capture role functioning should be explored. Finally, patients with HR MDS, CMML, or AML also experience other symptoms related to cytopenia, for example related to thrombocytopenia or neutropenia. Further research on an acceptable measurement approach to these symptoms would allow a more comprehensive understanding of the experience of patients with HR MDS, CMML, or AML.

We also acknowledge some limitations in the data collected for our online study. Firstly, the sample consisted of only 51 subjects. The RMT parameters could be estimated in this relatively small sample, with a reasonable amount of uncertainty, but our results should be confirmed in larger samples. Typically, the specific examination of the measures in each diagnosis subgroup (HR MDS, CMML, and AML) needs to be further explored to confirm the absence of major differences in responses of patients from a different diagnosis subgroup. It also would be relevant to explore whether the measure of anemia-related symptoms allows characterizing the disease continuum from MDS to AML in a larger sample including enough patients within each subgroup. This may show, for example, that patients with AML are more likely to report more severe anemia-related symptoms, such as dizziness. Secondly, our sample was a convenience sample, and all the variables were reported by patients, including their diagnosis. This raises questions about how representative this sample is of the population of patient with HR MDS, CMML, and AML and the extent to which our findings in terms of coverage of the physical functioning and fatigue continuums by our item selection, as well as the good fit of the selected items to the Rasch model, are generalizable. Replicating our analyses in a larger, better clinically defined sample would reinforce our findings. For example, having a sample where the QLQ-C30 and supplemental items are collected together with good quality hemoglobin level data would allow for exploration of the association between hemoglobin level and severity of fatigue or anemia-related symptoms, hence consolidating their validity. Similarly, the construct validity of the scores based on the QLQ-C30 and supplemental items could be consolidated in future research by exploring their association with other measures of fatigue and physical functioning. Finally, the study was cross-sectional, preventing the examination of the longitudinal measurement properties of the PRO measures, such as test-retest reliability, ability to detect change over time, or exploration of what constitutes a meaningful change in score. Thus, more data will be needed to confirm our positive early results on the appropriateness of these modular measures of physical functioning and fatigue in this context.

## Conclusion

The EORTC QLQ-C30 and the selection of items from the EORTC Item Library offer a promising pragmatic solution to measure physical functioning and key disease-related symptoms (fatigue and other anemia-related symptoms) in HR MDS, CMML, and AML. Specifically, the QLQ-C30 plus supplemental items could contribute to the demonstration of the benefit of new treatments in the context of clinical trials in this patient population.

## Supplementary Information

Additional file 1 .

Additional file 2. Supplemental materials: Item characteristic curves.

## Data Availability

For inquiries regarding the data set and possibilities of data sharing, please contact the authors.
