# Supplementary material for: Measuring patient-reported physical functioning and fatigue in myelodysplastic syndromes using a modular approach based on EORTC QLQ-C30
Source: J Patient Rep Outcomes. 2021 Jul 20;5:60. doi: 10.1186/s41687-021-00334-w (PMC8292469; doi:10.1186/s41687-021-00334-w)

# Supplemental materials: Item characteristic curves

## EORTC QLQ-C30 Physical functioning items

**Item “Trouble do strenuous activities”**


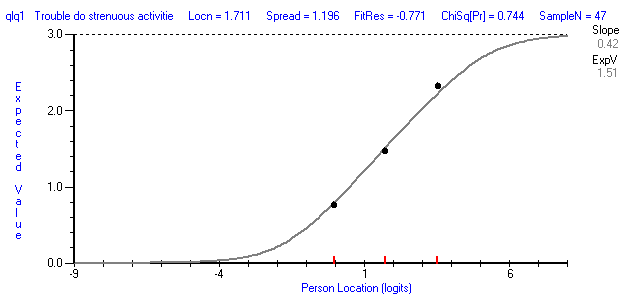


**Item “Trouble taking a long walk”**


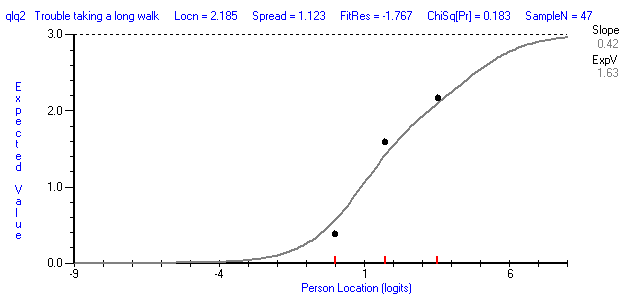


**Item “Trouble doing short walk out”**


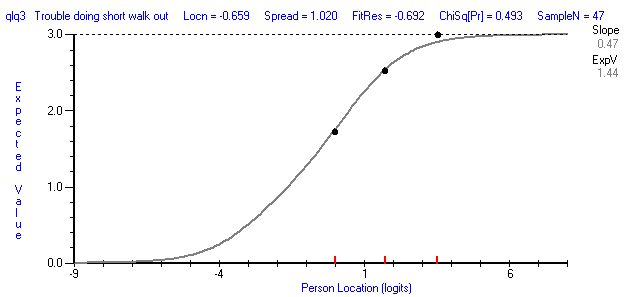


**Item “Need to stay in bed or chair”**


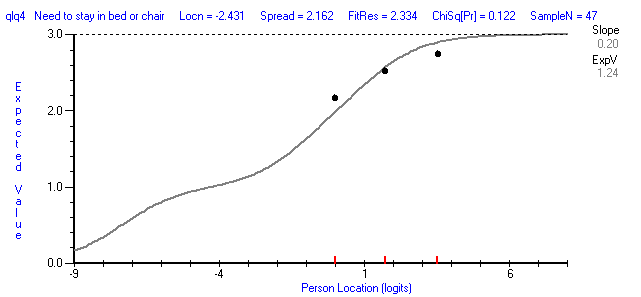


**Item “Need help with eat wash toilet”**


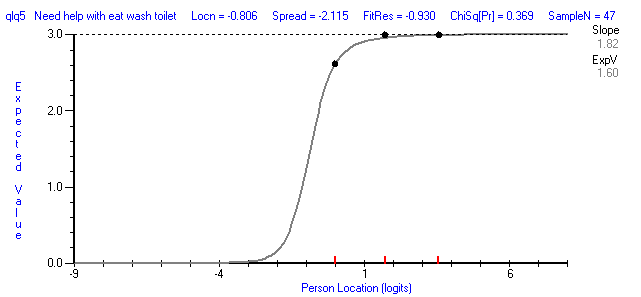


## EORTC QLQ-C30 Physical functioning and supplemental items

**Item “Trouble do strenuous activities”**


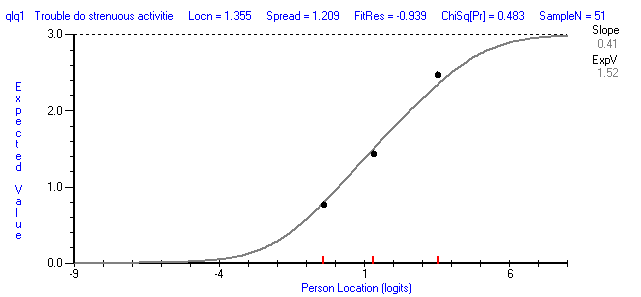


**Item “Trouble taking a long walk”**


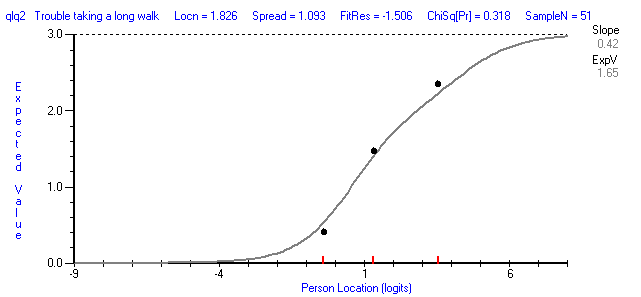


**Item “Trouble doing short walk out”**


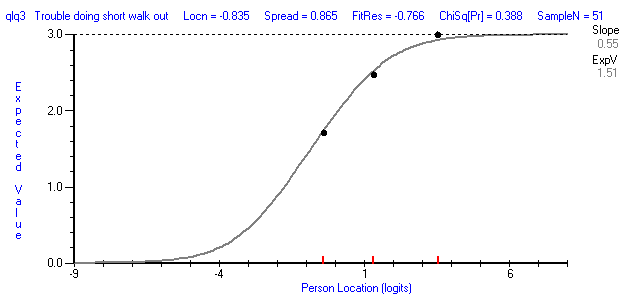


**Item “Need to stay in bed or chair”**


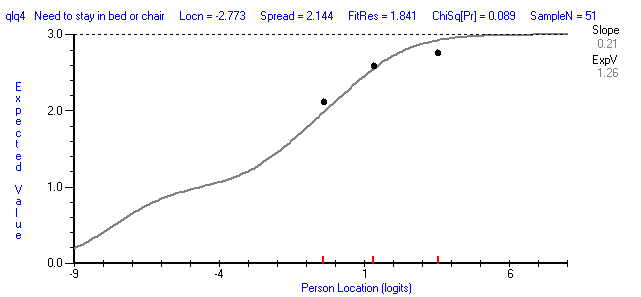


**Item “Need help with eat wash toilet”**


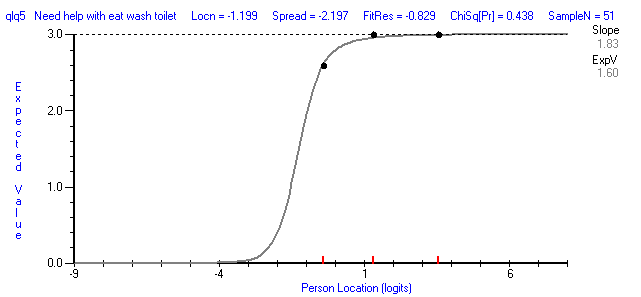


**Item “Slowed down”**


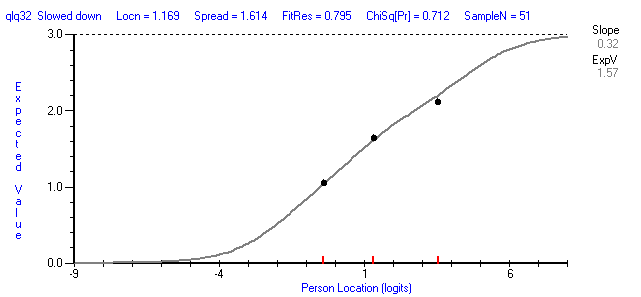


**Item “Difficulty climbing stairs”**


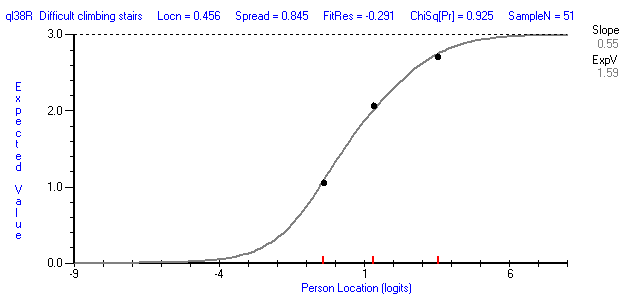


## EORTC QLQ-C30 Fatigue items

**Item “Need to rest”**


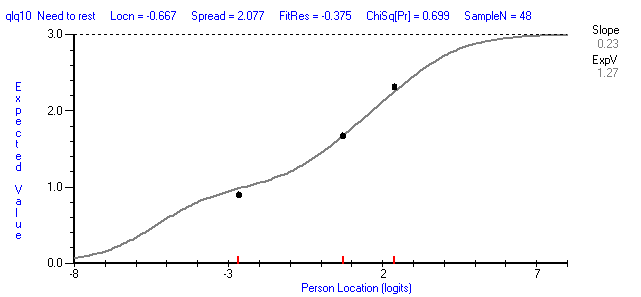


**Item “Felt weak”**


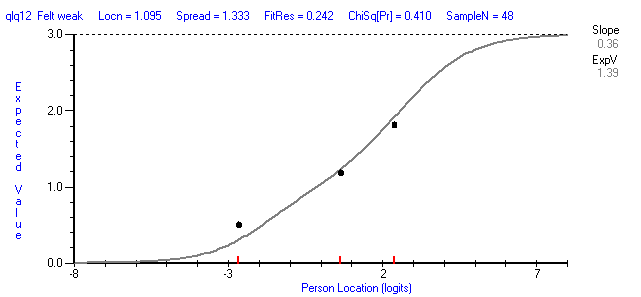


**Item “Were you tired”**


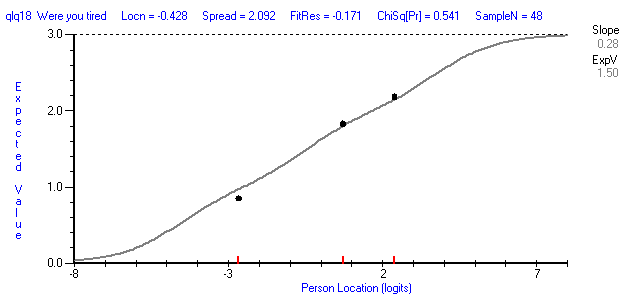


## **EORTC QLQ-C30 Fatigue and supplemental items**

**Item “Need to rest”**


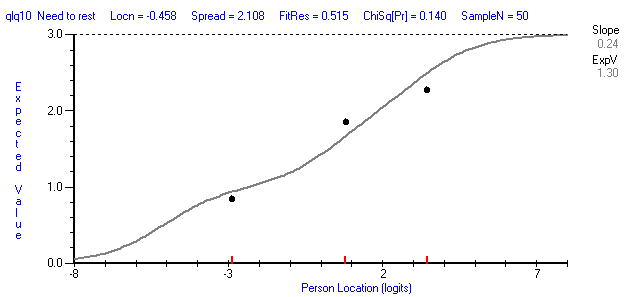


**Item “Felt weak”**


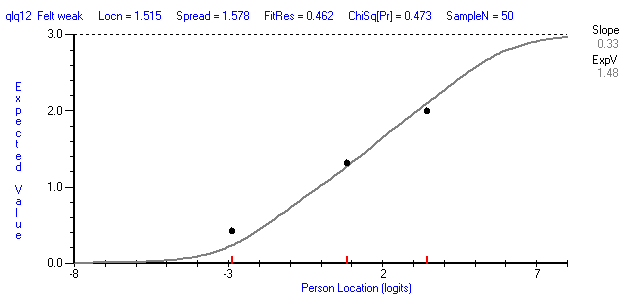


**Item “Were you tired”**


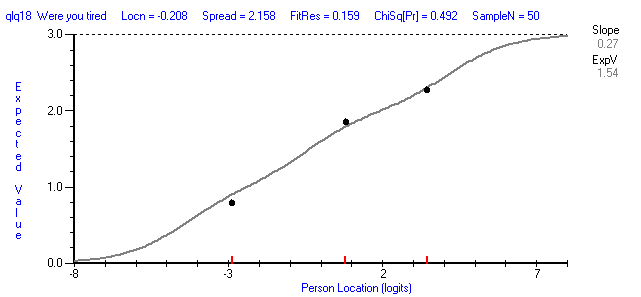


**Item “Weak in arms or legs”**


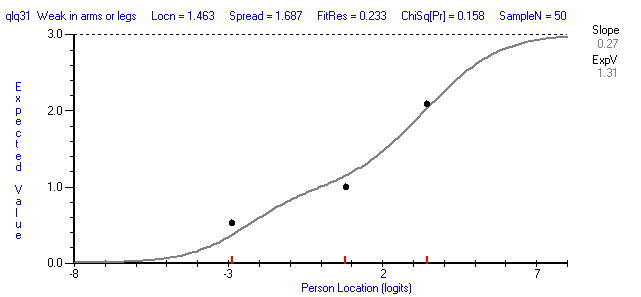


**Item “Become easily tired”**


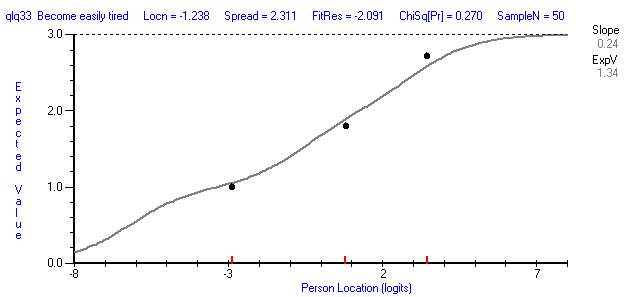


**Item “Lacked energy”**


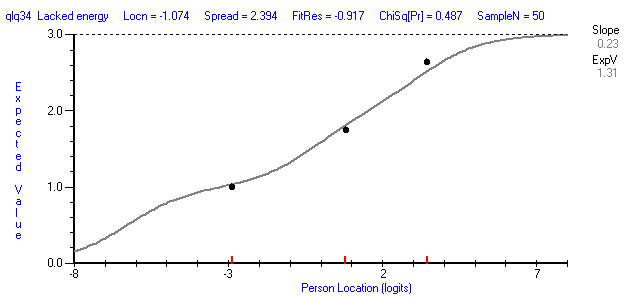


## **EORTC QLQ-C30 and supplemental items assessing fatigue and anemia-related symptoms**

**Item “Need to rest”**


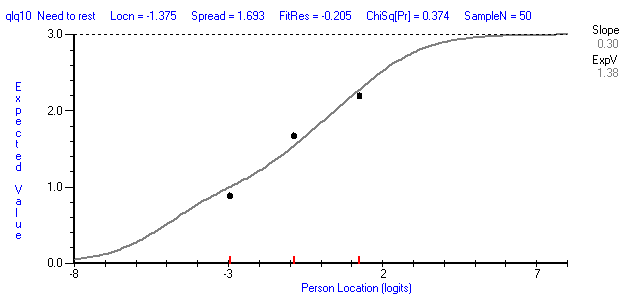


**Item “Felt weak”**


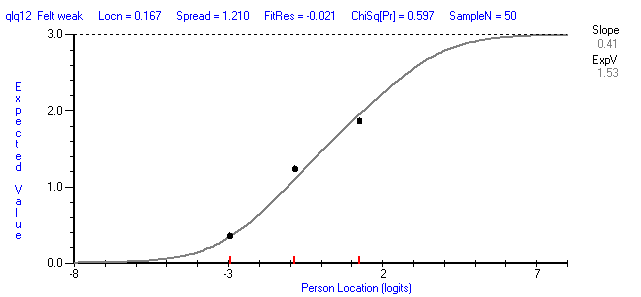


**Item “Were you tired”**


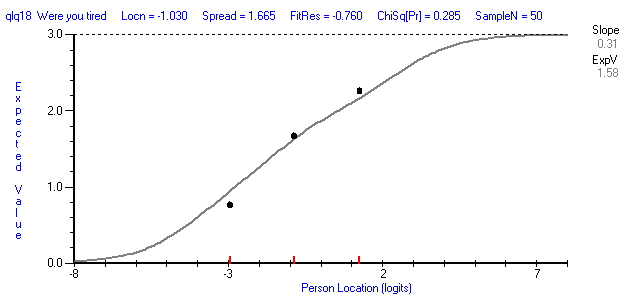


**Item “Weak in arms or legs”**


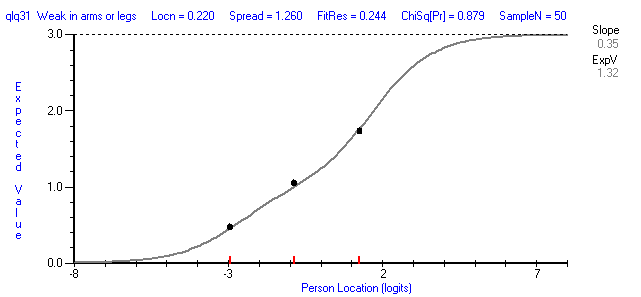


**Item “Become easily tired”**


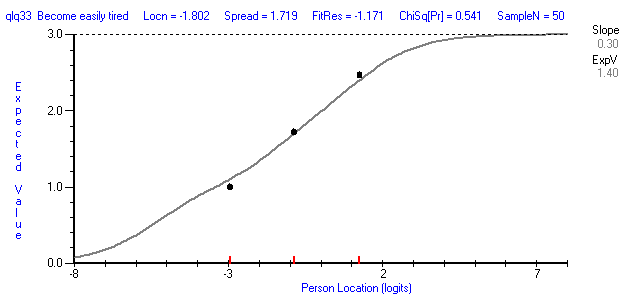


**Item “Lacked energy”**


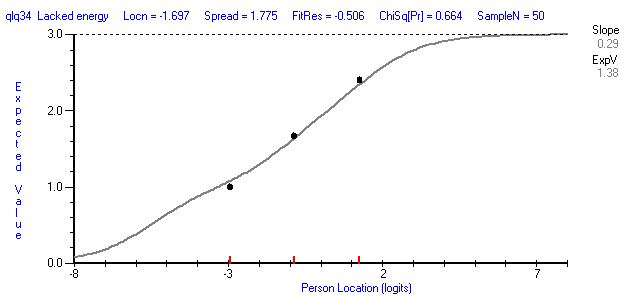


**Item “Were you short of breath”**


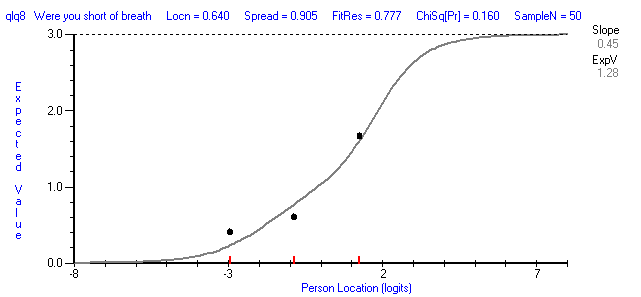


**Item “Shortness breath on exertion”**


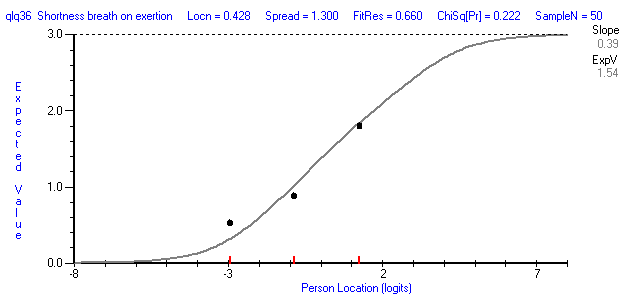


**Item “Stop for breath when walking”**


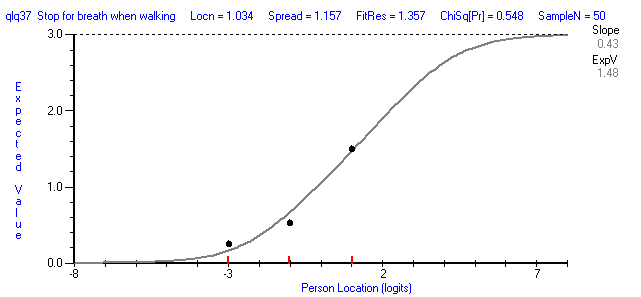


**Item “Been dizzy”**


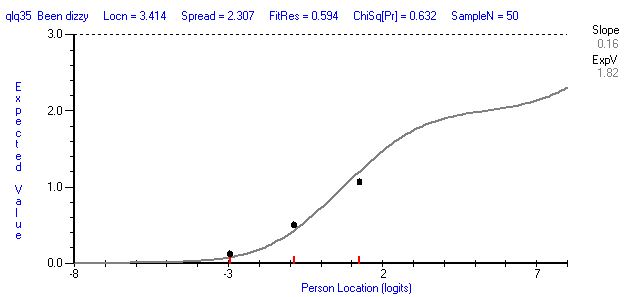

Supplement: Supplementary file 2 — Supplemental materials: Item characteristic curves. [file 41687_2021_334_MOESM2_ESM.docx]
